# Modern Medical Therapeutics

**Published:** 1885-07

**Authors:** 


**Modern Medical Therapeutics.** A Compendium of Recent Formulæ and Specific Therapeutical Directions, from the practice of eminent contemporary physicians—American and foreign. By GEORGE H. NAPHEYS. A.M., M.D. Edited by Drs. EDWARDS and BRINTON, Eighth edition. Enlarged and revised. Philadelphia: D. G. Brinton, 115 South Seventh street. 1885.

The author believes that "in this work the art of Therapeutics is presented in all its aspects, and divested of that barren theorizing which has been its bane;" whatever question there may be as to this statement, it is beyond dispute that books of this character have multiplied, probably in obedience to "a long felt want," and that the one before us is the best of its class. The present volume has been edited by the editors of the "Medical and Surgical Reporter," who have had unusual facilities for the work, and the result of their labors are evident throughout the volume, materially enhancing its value.

